# Working people with type 1 diabetes in the Finnish population

**DOI:** 10.1186/s12889-017-4723-8

**Published:** 2017-10-12

**Authors:** Pirjo Hakkarainen, Reijo Sund, Martti Arffman, Sari Koski, Vilma Hänninen, Leena Moilanen, Kimmo Räsänen

**Affiliations:** 1 School of Medicine, Institute of Public Health and Clinical Nutrition, University of Eastern Finland, Kuopio Campus, P.O. Box 1627, FI-70211 Kuopio, Finland; 2 Centre for Research Methods, Department of Social Research, University of Helsinki, Helsinki, Finland; 3 School of Medicine, Institute of Clinical Medicine, University of Eastern Finland, Kuopio, Finland; 4 Department of Health and Social Care Systems, National Institute for Health and Welfare, Helsinki, Finland; 5 The Finnish Diabetes Association, Tampere, Finland; 6 Department of Social Sciences, University of Eastern Finland, Kuopio, Finland; 7 Department of Medicine, Kuopio University Hospital, Kuopio, Finland

**Keywords:** Type 1 diabetes, Incidence, Prevalence, Employment, Work, Self-rated health, Work ability, Retirement, Occupational health care, Finland

## Abstract

**Background:**

The incidence of type 1 diabetes is increasing worldwide. Since so little is known about work life of individuals with type 1 diabetes, we examined incidence and prevalence trends of type 1 diabetes among working-aged Finns. We also investigated the employment rate and how workers with type 1 diabetes perceive their health and work ability, and their intended retirement age.

**Methods:**

We analyzed changes in the incidence, prevalence, and employment rate using nationwide multi-register-based FinDM data, and estimated a Self-Rated Health, Work Ability Score, and inquired about retirement intentions of 767 working individuals with type 1 diabetes in a cross-sectional survey. All estimates were compared to the corresponding data of the Finnish general population.

**Results:**

The average annual age-standardized incidence rate of type 1 diabetes among men aged 18–39 was 29 per 100,000/year; the incidence rate has increased by 33% from 1992 to 2007. Among women, the incidence remained at 16 per 100,000/year. Among working-aged (18–64) people, the age-standardized prevalence of type 1 diabetes increased by 39% among women and 33% among men. Two out of every three working aged individuals with type 1 diabetes were in the labor force; this is about 10% lower than in the Finnish population. The average age-standardized employment rate among those individuals with type 1 diabetes belonging to the labor force was 82%, compared to 84% in the general population. Working individuals with type 1 diabetes rated their health and work ability as being slightly lower than the general working population, but nonetheless, there were no significant differences in retirement intentions.

**Conclusions:**

Between 1992 and 2007, the number of working-aged people and workers with type 1 diabetes increased by 35%. Most workers with type 1 diabetes manage as well at work as the general population. Special attention should be paid to workers with type 1 diabetes when they are diagnosed and/or report moderate or poor work ability.

**Electronic supplementary material:**

The online version of this article (10.1186/s12889-017-4723-8) contains supplementary material, which is available to authorized users.

## Background

The incidence of type 1 diabetes has increased globally on average by 3–5% per year [1]. Finland has the highest rate of type 1 diabetes in the world [2, 3]. Between 2006−2011, the mean incidence of type 1 diabetes in children under 15 years was 62.5 per 100,000/year [4]. In all, there are about 50,000 people with type 1 diabetes in Finland, i.e. 0.9% of the total population [5, 6]. They represent about 15% of the prevalent diagnosed cases of diabetes in Finland [7].

In type 1 diabetes, the blood glucose level tends to be elevated. However, insuIin administration, which is used to regulate the blood glucose level, may predispose the individual to hypoglycaemia [8, 9]. A worker with type 1 diabetes has to ensure that his/her blood glucose level remains optimal during the working hours [9]. Optimal control of blood glucose levels is not only important in the short term, it is also necessary to avoid severe long-term pathological outcomes i.e. diabetic kidney and eye diseases, diabetic neuropathy and cardiovascular diseases, all of which exert a profound effect on an individual's health [8, 9].

The above-mentioned health-related issues including hypoglycaemia, hyperglycaemia and other complications can impair the individual’s functional capacity, have medical and financial consequences as well as reducing his/her social well-being and quality of life [10–13]. Even non-severe hypoglycaemic events can weaken a worker’s ability to manage his/her duties and require time off work [10]. In addition, it has been shown that severe hypoglycaemia and diabetes-related complications are associated with discrimination in the workplace [14] and early retirement [15]. Furthermore, these health-related complications have been a reason for changing the workplace, even unemployment [12, 16]. Finally, considerable overall excess mortality has been found in individuals with insulin-treated diabetes [17].

Some of the previous studies of employment among individuals with type 1 diabetes have suggested that individuals with type 1 diabetes may be less likely to be employed than the general population [13]. However, there is no consensus as there are some reports where the situation is opposite [18] and one report which found no difference between individuals with type 1 diabetes and the general population [19].

Type 1 diabetes is costly for the society. For instance, in the UK, direct healthcare costs of type 1 diabetes have been estimated to consume more than 1% of the healthcare budget [20]. Diabetes-related complications are responsible for a major proportion of direct health expenditures. In addition to direct healthcare costs, diabetes also exerts a major influence via indirect costs, mainly attributable to reduced productivity linked with premature death, early retirement, absenteeism and presenteeism [20, 21].

Little research has been conducted on the employment rate of people with type 1 diabetes and their work life [13, 22, 23], although this kind of information is very important to employers and societies when trying to keep a high proportion of the work force in work and postponing premature retirement.

In 2011, the number of working-aged people with type 1 diabetes in Finland was around 30,000 [National Institute for Health and Welfare (THL). Diabetes in Finland (FinDM), 2011. Unpublished database]. It is not known how many of them are working and how these numbers have changed in recent years. It is also not known how people with type 1 diabetes rate their health, work ability, and intended retirement age in comparison to the general working population.

First, we aimed to determine incidence and prevalence trends of type 1 diabetes among working-aged Finns. Next, we investigated the proportion of working-aged Finns with type 1 diabetes belonging to the labor force and calculated their employment rates in comparison with an aged-matched general Finnish population. Finally, we assessed how working Finns with type 1 diabetes perceive their health and work ability, and asked them about their intended retirement age which we compared to the general population’s.

## Methods

### Two national diabetes studies

This study is based on two national diabetes studies conducted in Finland. The FinDM database covers all people with diabetes since 1964. Individuals with type 1 diabetes were identified from several Finnish national registers [24]. We used a version of the database that was linked to employment data covering people with diabetes from 1964 until 2007. Data on the labor force and employment were obtained from the Employment Statistics maintained by Statistics Finland and individually linked to the FinDM data for the 1992–2007 period using the unique personal identification codes. Those belonging to the labor force were defined as employed or unemployed during the last week of the previous calendar year. Those not included in the labor force included students and pupils, pensioners, military conscripts and other special groups [25].

The “People with Type 1 Diabetes in Worklife” survey was implemented by the University of Eastern Finland and Kuopio University Hospital. This national survey was carried out by sending a postal questionnaire to a random sample of 2500 people with type 1 diabetes aged 18*–*65 years from the Medication Reimbursement Register of the Social Insurance Institution of Finland. Out of the sample of 2500, 1214 subjects with type 1 diabetes returned the form, a response rate of 49%.The data were collected cross-sectionally in 2010–2011. This project has been described elsewhere [26].

### Identifying people with type 1 diabetes

FinDM: On the basis of the register data, people with type 1 diabetes (insulin-dependent diabetes) were identified using data based on their need for antidiabetic medication. It was assumed that in practice all individuals with type 1 diabetes were under the age of 40 at the diagnosis date and had required constant insulin therapy since the diagnosis of diabetes, and that they did not use oral antidiabetic drugs intended to increase pancreatic insulin secretion [24].

The “People with Type 1 Diabetes in Worklife” survey: According to the Medication Reimbursement Register of the Social Insurance Institution of Finland, the participants in this survey had type 1 diabetes. If a participant confirmed having a type 1 diabetes diagnosis, he/she was accepted into the study.

### Measurements

“People with Type 1 Diabetes in Worklife” survey: the sociodemographic variables in the survey included gender, age, marital status, and education. The questions concerning diabetes queried the duration of diabetes (classified as 0–5, 6–10, 11–15, and >16 years), and last checked HbA1c (glycosylated hemoglobin) level (1 = ≤60 mmol/mol (≤7.5%); 2 = 61–70 mmol/mol (7.6–8.5%); 3 = 71–80 mmol/mol (8.6–9.5%); 4 = ≥81 mmol/mol (≥9.6%). In addition, the respondents were asked: “Have you had hypoglycemic events in which help has been needed during the last 12 months?” Severe hypoglycemic events in the past 12 months were rated on a scale of 0–2 (no; once; twice or more often).

### Self-rated health

Self-Rated Health (SRH) is one of the most frequently used measures of general health in epidemiological studies [27, 28]. SRH was assessed by asking the question “How would you assess your present state of health?”. Respondents rated their current perceived health on a scale of 1–5 (1 = good; 2 = reasonably good; 3 = average; 4 = rather poor; 5 = poor) [29]. The SRH of Finnish working individuals aged 18–64 years with type 1 diabetes was compared to the data of the working general population of the same age in Finland. The previously unpublished population-level comparison data of working Finnish people from 2010 was obtained for SRH from the National Institute for Health and Welfare [National Institute for Health and Welfare (THL). Health Behaviour and Health among the Finnish Adult Population, Spring 2010 survey. Unpublished database].

“Health Behaviour and Health among the Finnish Adult Population (AVTK)” was conducted by the National Institute for Health and Welfare. The survey monitored health and lifestyles among Finns and it is intended to follow-up changes in the health behaviour both at the level of populations and in distinct population groups. The survey has been carried out as a postal questionnaire in 1978−2014. The 2010 random sample (*N* = 5000) from the Population Register represented the whole Finnish working age population from 15 to 64 years. The response rate was 57% (*n* = 2826) [30].

### Self-rated work ability

Self-rated work ability was measured with the use of the Work Ability Score (WAS), which assesses current work ability compared to the lifetime best. The respondents were asked one question: “Assume that your work ability at its best has a value of 10 points. How many points would you give your current work ability?” This question is one item of the Work Ability Index (WAI) [31]. The scores (0–10) were categorized into four groups according to the recommendation of the designers of the WAI: 0–5 = poor; 6–7 = moderate; 8–9 = good; and 10 = excellent [32]. The WAS of Finnish working individuals aged 18–64 years with type 1 diabetes was compared with the data of the working general population in Finland of the same age. The population-level comparison WAS data of working Finnish people for the year 2009 was obtained from the Finnish Institute of Occupational Health [Finnish Institute of Occupational Health. Finnish National Work and Health survey, 2009. Unpublished database].

“Finnish National Work and Health” personal interview survey is conducted by the Finnish Institute of Occupational Health since 1997 [33]. The survey serves as a national surveillance system on perceived working conditions and the health of the working-age population. The random sample (*N* = 6000) from the Statistics Finland (the Employment Statistics) represents the whole Finnish working age population from 20 to 64 years of age (in 2009). The Finnish Institute of Occupational Health has limited the survey only to those who were working (*n* = 4516). The response rate was 58% (*n* = 2614) [Finnish Institute of Occupational Health. Finnish National Work and Health survey, 2009. Unpublished database].

### Retirement intentions

Retirement intentions were inquired from wage-earners aged 45–64 years with the question “At what age do you reckon you will retire on full-time pension?” [34]. The respondents informed the age in years. The years were categorized into two groups according to an old-age pension legislation in Finland (1 = 18–62; 2 = 63–) [35]. The retirement intentions of Finnish wage-earners aged 45–64 years with type 1 diabetes were compared with the data of the wage-earning population in Finland of the same age. The population-level comparison data of retirement intentions of Finnish wage-earners for 2008 were obtained from Statistics Finland [Official Statistics of Finland (OSF). Quality of work life survey, 2008. Unpublished database].

“Quality of work life” survey is a personal interview survey conducted by the Statistics Finland since 1977. The survey monitors working conditions including work environments, contents of work, labor market positions and conditions of employment. The sample (*N* = 6500) from the Statistics Finland (the Employment Statistics) represents the whole Finnish entire wage and salary earning population from 15 to 64 years of age (in 2008). The response rate was 68% (*n* = 4392) [34].

### Statistics

**Fig. 1:**
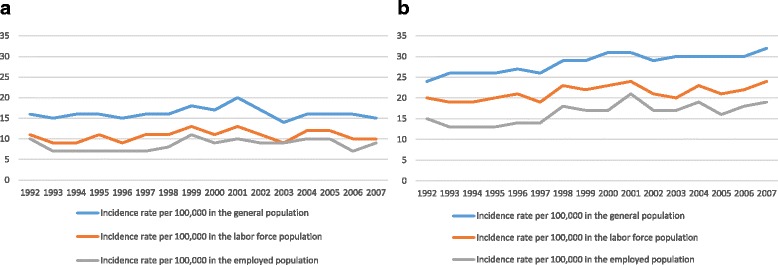
Age-standardized incidence of type 1 diabetes among Finns aged 18–39 years in 1992–2007. **a** Women; **b** Men

**Fig. 2:**
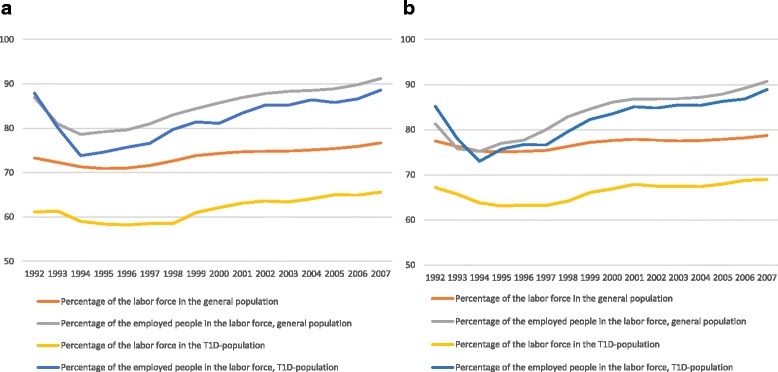
Age-standardized percentage of 1) people belonging to the labor force in the general population, 2) employed people in the labor force in the general population, 3) people belonging to the labor force in T1D-population and 4) employed people in the labor force in T1D-population in 1992–2007. **a** Women; **b** Men. T1D = Type 1 diabetes

We used the FinDM database to assess changes in the incidence and prevalence estimates of type 1 diabetes between 1992 and 2007. To derive the incidences, we calculated the numbers of new cases of type 1 diabetes each year and divided those numbers by the corresponding follow-up time measured in person-years in the risk population (Fig. 1). An additional file shows this in more detail (see Additional file 1: Table S1). Prevalence was calculated by dividing the average number of people with type 1 diabetes alive during the year by the number of the corresponding total population. Annual age-standardized rates with 95% confidence intervals (CI) were computed using the direct method of standardization, with the European Standard Population as the standard population in 1992–2007 (Fig. 2). An additional file shows this in more detail (see Additional file 2: Table S2). We derived the incidence and prevalence figures using stratification by employment status.

A similar standardization method was used to calculate the age-standardized proportions of working-aged individuals with type 1 diabetes and, correspondingly, percentages of the labor force with type 1 diabetes and employed individuals with type 1 diabetes among the working-age general population. The numbers for the general population were obtained from Statistics Finland.

**Fig. 3:**
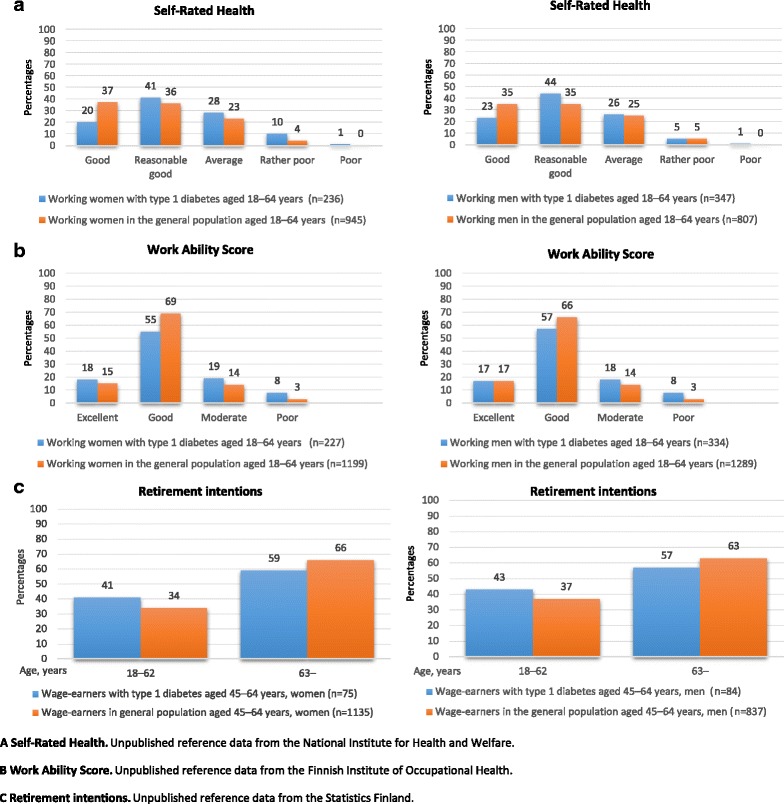
Self-rated health, work ability and retirement intentions among working women and men with type 1 diabetes and the general population. **a** Self-Rated Health; **b** Work Ability Score; **c** Retirement intentions

The frequencies, percentages, means, standard deviation (SD) and range were used to describe characteristics of the participants of the “People with Type 1 Diabetes in Worklife” survey. An additional file provides more details [See Additional file 3: Table S3]. The frequencies and percentages of SRH and WAS were calculated separately for working men and women (Fig. 3). The frequencies and percentages of retirement intentions were calculated only for 45–64-year-old wage-earning men and women (Fig. 3). The statistical significance of the difference of distributions was assessed by chi-squared tests and Fisher’s exact test. Statistical analyses were carried out in SPSS for Windows, Rel. 21.0.0.1. 2016 (SPSS Inc., Chicago, IL, USA), and SAS Rel. 9.3 (SAS Institute, Cary, NC, USA) and in R, version 3.3.1.

## Results

### Incidence and prevalence of type 1 diabetes among working-aged individuals in Finland in 1992–2007

The average annual age-standardized incidence rate of type 1 diabetes among women aged 18–39 years was 16 per 100,000 per year, and it remained at the same level throughout the 1992–2007 period (Fig. 1). The average annual age-standardized incidence rate of type 1 diabetes among men aged 18–39 years was 29 per 100,000 per year, increasing from 24 (95% CI 21–28) per 100,000 per year in 1992 to 32 (95% CI 28–37) per 100,000 per year in 2007. This was an increase of 33% over the 15-year period. On average, 71% of 18–39 aged men and women with incident type 1 diabetes (i.e. who were initially diagnosed with diabetes within this age range) belonged to the labor force when diagnosed and 79% of incident cases belonging to labor force were employed (Fig. 1). An additional file shows this in more detail [See Additional file 1: Table S1].

The prevalence of type 1 diabetes among working-aged (18–64 years) people was 541 per 100,000 (95% CI, 533–549) in 1992 and 732 per 100,000 (95% CI, 722–741) in 2007, representing an increase of 35% over the 15-year period. Among women, the age-standardized prevalence of type 1 diabetes increased from 449 (95% CI 438–459) per 100,000 in 1992 to 622 (95% CI 610–634) per 100,000 in 2007, an increase of 39%. Among men, the rates were somewhat higher: 631 (95% CI 619–643) in 1992 and 839 (95% CI 825–853) in 2007, but the numerical increase was less i.e. 33%. [See Additional file 2: Table S2].

### Belonging to the labor force and employment

Between 1992 and 2007, the proportion of people with type 1 diabetes belonging to the labor force fluctuated between 58% and 66% for working-aged women and 63% and 69% for working-aged men (Fig. 2). The mean value of age-standardized proportions was about 10 percentage points lower than the corresponding range in the general population.

During the same period, on average a total of 82% of individuals with type 1 diabetes belonging to the labor force were actually employed (Fig. 2). This was very similar to the value of 84% of individuals in the general population belonging to labor force who were gainfully employed. Employment percentages fell to their minimum in 1994 but after that date, the percentages rose. Among those with type 1 diabetes belonging to the labor force, 89% of individuals were employed in 2007 whereas 91% of individuals of the general population in the labor force were employed. Between 1992 and 2007, the total number of employed individuals with type 1 diabetes increased by 38% among women and by 34% among men (Fig. 2). An additional file shows this in more detail [See Additional file 2: Table S2].

### Self-rated health, work ability and intended retirement age

A total of 767 individuals who had been diagnosed with type 1 diabetes and who had been working during the past 12 months participated in the “People with Type 1 Diabetes in Worklife” survey. The characteristics of the participants are shown in Additional file 3: Table S3.

A total of 20% of working women with type 1 diabetes rated their health as good; this is almost half the corresponding value (37%) of working women in the general population (Fig. 3). Working women with type 1 diabetes were more likely to rate their health slightly more often as reasonably good, average, rather poor, or poor than working women in the general population (*p* < 0.001). Working men with type 1 diabetes also rated their health as good less frequently than working men in the general population. However, they reported reasonably good and average health more often than working men in the general population (*p* < 0.01). The difference between men and women with type 1 diabetes was statistically almost significant (*p = 0.083*).

A total of 18% of working women with type 1 diabetes and 15% of working women in the general population rated their work ability as excellent (Fig. 3). Working women in the general population were more likely to assess their work ability as good (69%) than working women with type 1 diabetes (55%), with the latter reporting moderate or poor work ability more often (*p* < 0.001). The figures were similar among the men (*p* < 0.001). There was no significant difference between men and women with type 1 diabetes in self-rated work ability.

When the assessment was restricted to 45–64-year-old wage-earners, a total of 41% of female wage-earners with type 1 diabetes and 34% of female wage-earners in the general population intended to retire before the age of 63 years (*p* = *NS*), (Fig. 3). Similarly, 43% of male wage-earners with type 1 diabetes intended to retire before the age of 63 years, a value that was only slightly greater than the 37% of wage-earning men in the general population (*p* = *NS*). In general, most of the wage-earners intended to retire at the age of 63, although about 20% of the 45–64-year-old wage-earners intended to retire at the age of 60. There was no statistically significant difference between men and women with type 1 diabetes in their intended retirement age.

## Discussion

This nationwide study reports register-based data on the incidence, prevalence, and employment of working-aged (18–64 years) Finns with type 1 diabetes as well as survey data on how working individuals with type 1 diabetes perceive their health, work ability and intended retirement age. The results were compared to the general working-age population in Finland.

### Incidence of type 1 diabetes among people aged 18–39 years

There is a paucity of knowledge of the incidence rates of type 1 diabetes among working-aged people and therefore we studied incidence in detail. The age-standardized incidence rates of type 1 diabetes were higher among men aged 18–39 years compared to women of the same age. The rates increased by 33% in men over the 15-year period, while there was no increase among women. This result corresponds to an earlier study on young adult Finns aged 15–39 years, which identified a high incidence of type 1 diabetes and a predominance of men in these young adults [36]. A predominance of males has also been found among children under 15 years with type 1 diabetes in Finland [2, 4, 37]. Thus, a predominance of males seems to be present in various age groups with type 1 diabetes.

Among individuals with type 1 diabetes aged 18–39 years, almost 80% of those belonging to the labor force were already in paid employment when they were diagnosed with type 1 diabetes. As far as we are aware, this is the first investigation into the employment status of individuals when they were diagnosed with type 1 diabetes. It may be challenging to cope at work when newly diagnosed with type 1 diabetes. There is evidence that individuals with type 1 diabetes experience diabetes distress [38–40] and work-related diabetes distress [26], which might impact on their metabolic control. Trying to combine work and type 1 diabetes might be especially challenging when an individual is learning to self-manage diabetes. These challenges should be studied further and appropriate measures of support should be developed for working individuals with recently diagnosed type 1 diabetes.

### Prevalence of type 1 diabetes among people aged 18–64 years

We found that during the 15-year period examined, the age-standardized prevalence of type 1 diabetes increased by 39% among working-aged (18–64 years) women and 33% among working-aged men i.e. the increase in prevalence was relatively lower in men. This result contradicted our finding that incidence rates have been increasing among men aged 18–39 years, but not among women of the same age. We suggest that associations between the prevalence of type 1 diabetes and the possible causes of the decrease in prevalence rates at working age – such as death – among working-aged individuals should be investigated.

While the life expectancy of individuals with type 1 diabetes has been estimated in some countries e.g. in Scotland and Sweden (loss of life expectancy = approximately 10–13 years) [41, 42], it has not been investigated in Finland. However, considerable overall excess mortality has been reported in insulin-treated Finns [17].

Overall, it is difficult to compare the prevalence rates of type 1 diabetes among working-aged individuals to other studies since some of them have reported the prevalence of type 1 diabetes among children [43], adolescents [44], or the entire population [45], while others have not separated prevalence into type 1 and type 2 diabetes [46].

### Employment

We showed that roughly two out of every three working-aged individuals with type 1 diabetes belonged to the labor force, a value about 10 percentage points less than in general population of Finland. We suggest that clarifying the causes of this gap would be an important topic for further research.

We found that employment rates were at the same level among the labor force with type 1 diabetes and the general population. In Finland, this is in harmony with the Non-discrimination Act (1325/2014) [47], which promotes equality in working life e.g. no discrimination due to a health condition. Thus, workers with type 1 diabetes should not be discriminated against because of their diabetes. Unemployment rates were highest (18-20%) among the labor force in Finland in 1992–1994. With recovery from the economic recession, there was a trend towards greater employment, year on year until 2007, when the unemployment rate was about 7% [48]. A similar trend was seen among individuals with type 1 diabetes.

As said, labor legislation supports equality and we found no differences in unemployment rates between the general population and people with type 1 diabetes. However, a fear of discrimination at work, which might damage their career prospects, may increase the likelihood of the workers with type 1 diabetes to conceal their condition and health status [49, 50]. Thus, concealment of type 1 diabetes in working life needs to be studied further.

Previous results of employment among individuals with type 1 diabetes are sparse and conflicting. A previous cross-sectional study from Netherlands showed that there were similar unemployment rates among employees with type 1 diabetes as in the general Dutch population [19]. However, a novel cross-sectional study from Denmark, based on two surveys, has revealed that individuals with type 1 diabetes are more frequently unemployed than the general population of Denmark [13]. In contrast, a repeated cross-sectional study from U.S. found that women aged 18–65 years with type 1 diabetes were more likely to be employed than women without diabetes [18]. This is at odds with a large-scale follow-up study from Sweden, which stated that women aged 19–38 years with type 1 diabetes were less likely to be employed than their peers in the general population [51]. As far as is known, there are no previous register-based follow-up studies comparing employment rates between the whole population with type 1 diabetes and the general population.

### Self-rated health

After excluding people who do not belong to the labor force, employment among the type 1 diabetes population is at the same level as in the general population, but there may nevertheless be work-related issues linked to type 1 diabetes. In our study, the majority of working women and men with type 1 diabetes rated their health as reasonably good. Men with type 1 diabetes were more frequently likely to report better health than women with type 1 diabetes. Similar results have been found previously in two studies among adults with type 1 diabetes in the U.S. [52, 53]. We revealed a contradiction in incidence and prevalence rates between women and men with type 1 diabetes. In our study, the incidence of type 1 diabetes was higher among men than women in the 1992–2007 period. However, the prevalence of type 1 diabetes increased less among men than among women. It is possible that those men with type 1 diabetes who feel that their health is poor leave working life earlier than women.

Overall, women and men with type 1 diabetes rated their health as good less frequently than women and men in the general Finnish population. This is not surprising, given their chronic condition.

### Work ability

We assessed self-rated work ability by applying the Work Ability Score (WAS). There was no gender difference in WAS rates among workers with type 1 diabetes and the general population. We have reported previously the same result by using the Work Ability Index (WAI) among Finnish workers with type 1 diabetes [23]. Earlier evidence has demonstrated that this single-item question associates strongly with the complete WAI [54] and our findings confirm this proposal among workers with type 1 diabetes. To the best of our knowledge, there are no other studies reporting WAS or WAI among working-aged individuals with type 1 diabetes. Overall, the prevalence of type 1 diabetes has increased among workers in Finland, and workers with type 1 diabetes reported moderate and poor work ability slightly more often than the general population. A reduced work ability has been found to increase the risk of long-term sick leaves, disability pension, and unemployment [55]. In addition, poor work ability and poor general health increase the risk for dismissal and long-term unemployment after being made redundant [56]. Thus, special attention should be paid to those workers with type 1 diabetes that report moderate or poor work ability.

### Retirement intentions

An interesting finding was that there was no significant difference in retirement intentions between wage-earners with type 1 diabetes and the general population. In both groups, the most frequent (mode) intended retirement age was 63 years. In Finland, at the time of the data collection of this study people could retire flexibly on an old-age pension between the ages of 63 and 68. In addition, a disability pension can be granted for individuals between the ages of 18 and 62 if their work capacity has been reduced for at least 12 months due to an illness, a defect or a handicap. A full disability pension can be awarded if loss of work capacity is at least 60%. In less severe cases i.e. work capacity reduced by at least 40%, then a partial disability pension can be granted. The disability pension amounts to only about 60% of an old age pension [35] i.e. there is no financial incentive for early retirement. As far as we are aware, this is the first investigation of the retirement intentions of workers or wage-earners with type 1 diabetes. However, evidence from Brazil claimed that 4.2% of Brazilian working men and women with type 1 diabetes had retired prematurely due to disabilities [15]. In addition, a study from the Netherlands – which did not differentiate between the types of diabetes – showed that workers with diabetes from 11 European countries had an increased probability of receiving disability benefits and retiring early. Furthermore, work-related factors, such as high job demands with low job control or low rewards, increased the probability of early retirement among individuals with diabetes [57].

### Strengths and limitations

The main strengths of this study were the data covering the whole Finnish population with type 1 diabetes and the whole general population from several nationwide registers, the explicit identification of individuals with type 1 diabetes, and the long follow-up time (1992–2007). Finland is a country which houses valid population-based health and employment registers and these are widely used in epidemiological research [35, 37, 48, 58, 59]. Furthermore, there are comprehensive statistics on work disability factors such as illnesses, days of absence due to illness [60], and disability pensions [35]. As a limitation, these registries do not include any information on individual experiences. Thus, we complemented register-based information with the national surveys.

We wanted to describe the greater picture by showing how people with type 1 diabetes are employed in relation to the general population and how they manage at work. Among the key strengths of our survey were the random sample of 2500 working-aged people with type 1 diabetes, and the large representative national sample size of 767 working respondents with type 1 diabetes. The sample represented workers from a wide range of different organizations and types of work. The response rate for our survey was 49%, which can be considered as acceptable [61]. However, selection bias is a typical phenomenon in these kinds of surveys. In our survey, the distributions of age and living areas were similar between respondents and non-respondents, although women were slightly overrepresented in the sample. Thus, the sample seems to be truly representative of working-aged Finns with type 1 diabetes. However, it cannot be ruled out that the sample is biased according to some unknown variables, which affect the readiness to participate, e.g. health status and living habits.

The response rates for the population-level comparison data “Health Behaviour and Health among the Finnish Adult Population” (57%), “Finnish National Work and Health” (58%), and “Quality of work life” (68%) were acceptable, but the above-mentioned possibilities for bias may also impact on their validity.

Self-reported questionnaires are commonly used to gather research data and can be very advantageous in assessing issues that would be difficult to evaluate with interviews or objective measurements. We included the widely used SRH, WAS, and retirement questions in our survey. The questions were identically phrased in our “People with Type 1 Diabetes in Worklife” survey and in the Finnish comparison data. The respondents answered the same questions within a relatively short time period, which increases the validity of the comparison between the populations. Self-reported health and work ability have been found to be associated significantly with objective measurements of health, work ability, and early retirement [27, 28, 54].

The present study has some limitations. First, it can be challenging to distinguish individuals with type 1 diabetes from individuals with type 2 diabetes of working-age, so we cannot be completely sure that our classification which was based on the use of anti-diabetic medication and age represents a clinically verified diagnosis. Nonetheless, we are confident that the cases that we identified are actually being medically-treated as insulin-dependent patients, so this should not represent a major limitation. We suggest that further register-based follow-up studies are warranted in other countries because our findings can be generalized only to Western countries with similar kinds of labor and health care systems. We also investigated self-rated health and work ability as well as retirement intentions in a cross-sectional survey but this setting does not allow the causes and effects to be evaluated. In addition, all of the measurements used were self-reported, so recall bias may have influenced the results.

## Conclusions

In conclusion, the number of working-aged people and workers with type 1 diabetes has clearly increased, and their self-reported general health and work ability is rated slightly lower than that of the general population. There was no significant difference in the retirement intentions of 45–64-year-olds with type 1 diabetes and the general population of the same age. Thus, it is concluded that most of the workers with type 1 diabetes seem to manage at work as well as the general population. However, special attention should be paid to those individuals who are working when they are diagnosed with type 1 diabetes and those individuals with type 1 diabetes reporting moderate or poor work ability. It might be that serious problems at work accumulate in some workers with type 1 diabetes due to their condition. More expertise in health care may be required to identify workers who need special support as type 1 diabetes becomes more frequent in the workplace.

## Additional files

Additional file 1: Table S1. Incidence of type 1 diabetes among Finns aged 18–39 years in 1992–2007. Annual numbers for incidence of type 1 diabetes among Finns aged 18–39 years in 1992–2007. (DOCX 18 kb)

Additional file 2: Table S2. Working aged (18–64 years) general Finnish population, and age-standardized prevalence and proportion of type 1 diabetes among working aged individuals, labor force and employed individuals in Finland in 1992–2007. Annual numbers for working aged (18–64 years) general Finnish population, and age-standardized prevalence and proportion of type 1 diabetes among working aged individuals, labor force and employed individuals in Finland in 1992–2007. (DOCX 18 kb)

Additional file 3: Table S3. Characteristics of the participants in the “People with Type 1 Diabetes in Worklife” survey. (DOCX 14 kb)

## Abbreviations

CI: Confidence interval
HbA1c: Glycosylated hemoglobin
SD: Standard deviation
SRH: Self-Rated Health
WAI: Work Ability Index
WAS: Work Ability Score
