# Supplementary material for: Working people with type 1 diabetes in the Finnish population
Source: BMC Public Health. 2017 Oct 12;17:805. doi: 10.1186/s12889-017-4723-8 (PMC5639775; doi:10.1186/s12889-017-4723-8)
Supplement: Supplementary file 1 — Incidence of type 1 diabetes among Finns aged 18–39 years in 1992–2007. Annual numbers for incidence of type 1 diabetes among Finns aged 18–39 years in 1992–2007. (DOCX 18 kb) [file 12889_2017_4723_MOESM1_ESM.docx]

Additional file 1: Table S1. Incidence of type 1 diabetes among Finns aged 18–39 years in 1992–2007

| Year | Incident cases of type 1 diabetes among people aged 18–39 years | | Belonging to labor force when diagnosed with type 1 diabetes | | | Being employed when diagnosed with type 1 diabetes | | |
| --- | --- | --- | --- | --- | --- | --- | --- | --- |
|  | (N) | Age-standardized incidence rate per  100,000 in the general population | (N) | Age-standardized percentage of incident cases belonging to the labor force | Age-standardized incidence rate per  100,000 in the labor force population | (N) | Age-standardized percentage of employed among incident cases in the labor force | Age-standardized incidence rate per  100,000 in the employed population |
| Women |  |  |  |  |  |  |  |  |
| 1992 | 125 | 16 | 88 | 70.7 | 11 | 75 | 83.2 | 10 |
| 1993 | 114 | 15 | 74 | 65.4 | 9 | 59 | 73.8 | 7 |
| 1994 | 122 | 16 | 70 | 61.5 | 9 | 57 | 65.8 | 7 |
| 1995 | 118 | 16 | 86 | 73.0 | 11 | 57 | 67.7 | 7 |
| 1996 | 111 | 15 | 72 | 66.4 | 9 | 55 | 71.5 | 7 |
| 1997 | 118 | 16 | 82 | 69.5 | 11 | 55 | 72.2 | 7 |
| 1998 | 121 | 16 | 82 | 67.9 | 11 | 62 | 75.2 | 8 |
| 1999 | 136 | 18 | 99 | 68.3 | 13 | 84 | 79.6 | 11 |
| 2000 | 127 | 17 | 82 | 64.0 | 11 | 64 | 80.4 | 9 |
| 2001 | 142 | 20 | 95 | 64.9 | 13 | 71 | 83.1 | 10 |
| 2002 | 125 | 17 | 77 | 63.9 | 11 | 66 | 83.6 | 9 |
| 2003 | 102 | 14 | 67 | 70.0 | 9 | 62 | 84.3 | 9 |
| 2004 | 111 | 16 | 83 | 76.9 | 12 | 74 | 85.7 | 10 |
| 2005 | 115 | 16 | 83 | 73.4 | 12 | 69 | 85.5 | 10 |
| 2006 | 109 | 16 | 69 | 64.1 | 10 | 52 | 86.9 | 7 |
| 2007 | 105 | 15 | 73 | 69.7 | 10 | 62 | 89.0 | 9 |
| Men |  |  |  |  |  |  |  |  |
| 1992 | 201 | 24 | 168 | 81.8 | 20 | 125 | 78.8 | 15 |
| 1993 | 207 | 26 | 155 | 73.8 | 19 | 106 | 69.1 | 13 |
| 1994 | 212 | 26 | 153 | 69.4 | 19 | 105 | 62.8 | 13 |
| 1995 | 209 | 26 | 164 | 77.5 | 20 | 106 | 67.4 | 13 |
| 1996 | 210 | 27 | 167 | 75.5 | 21 | 116 | 69.8 | 14 |
| 1997 | 206 | 26 | 152 | 72.4 | 19 | 114 | 71.4 | 14 |
| 1998 | 225 | 29 | 182 | 78.8 | 23 | 143 | 77.9 | 18 |
| 1999 | 225 | 29 | 168 | 74.3 | 22 | 133 | 80.8 | 17 |
| 2000 | 237 | 31 | 175 | 72.4 | 23 | 127 | 82.2 | 17 |
| 2001 | 232 | 31 | 181 | 75.9 | 24 | 160 | 84.5 | 21 |
| 2002 | 222 | 29 | 157 | 70.7 | 21 | 126 | 84.3 | 17 |
| 2003 | 223 | 30 | 153 | 68.1 | 20 | 128 | 84.4 | 17 |
| 2004 | 227 | 30 | 172 | 73.6 | 23 | 143 | 84.5 | 19 |
| 2005 | 223 | 30 | 158 | 69.4 | 21 | 122 | 86.0 | 16 |
| 2006 | 224 | 30 | 160 | 71.6 | 22 | 133 | 86.5 | 18 |
| 2007 | 240 | 32 | 172 | 72.6 | 24 | 143 | 88.7 | 19 |
