# Supplementary material for: Working people with type 1 diabetes in the Finnish population
Source: BMC Public Health. 2017 Oct 12;17:805. doi: 10.1186/s12889-017-4723-8 (PMC5639775; doi:10.1186/s12889-017-4723-8)
Supplement: Supplementary file 2 — Working aged (18–64 years) general Finnish population, and age-standardized prevalence and proportion of type 1 diabetes among working aged individuals, labor force and employed individuals in Finland in 1992–2007. Annual numbers for working aged (18–64 years) general Finnish population, and age-standardized prevalence and proportion of type 1 diabetes among working aged individuals, labor force and employed individuals in Finland in 1992–2007. (DOCX 18 kb) [file 12889_2017_4723_MOESM2_ESM.docx]

Additional file 2: Table S2. Working aged (18–64 years) general Finnish population, and age-standardized prevalence and proportion of type 1 diabetes among working aged individuals, labor force and employed individuals in Finland in 1992–2007

|  |  | | | Age-standardized prevalence and age-standardized proportion | | | | | | | |
| --- | --- | --- | --- | --- | --- | --- | --- | --- | --- | --- | --- |
| Year | Working‑age general population | | | Working‑aged people with type 1 diabetes | | Labor force with type 1 diabetes | | | Employed with type 1 diabetes | | |
|  | (N) | Percentage of people belonging to the labor force in the general population | Percentage of employed people from those belonging to the labor force in the general population | (N) | Prevalence per 100,000 in the general population | (N) | Percentage of people belonging to the labor force in people with type 1 diabetes | Prevalence per 100,000 in labor force population | (N) | Percentage of employed people from those belonging to the labor force in people with type 1 diabetes | Prevalence per 100,000 in the employed population |
| Women |  |  |  |  |  |  |  |  |  |  |  |
| 1992 | 1581345 | 73.3 | 86.9 | 7196 | 449 | 4899 | 61.1 | 300 | 4305 | 87.9 | 262 |
| 1993 | 1585231 | 72.3 | 81.0 | 7356 | 458 | 5001 | 61.3 | 306 | 4031 | 80.2 | 245 |
| 1994 | 1588790 | 71.3 | 78.6 | 7534 | 469 | 4951 | 59.0 | 302 | 3722 | 73.8 | 225 |
| 1995 | 1591903 | 70.9 | 79.2 | 7731 | 481 | 5001 | 58.4 | 305 | 3813 | 74.6 | 231 |
| 1996 | 1595458 | 71.0 | 79.6 | 7910 | 493 | 5068 | 58.2 | 310 | 3914 | 75.7 | 238 |
| 1997 | 1599872 | 71.6 | 81.0 | 8073 | 504 | 5165 | 58.5 | 317 | 4019 | 76.6 | 245 |
| 1998 | 1605429 | 72.6 | 83.0 | 8239 | 514 | 5243 | 58.5 | 323 | 4194 | 79.7 | 257 |
| 1999 | 1610865 | 73.8 | 84.4 | 8431 | 526 | 5544 | 61.0 | 343 | 4543 | 81.4 | 280 |
| 2000 | 1615600 | 74.3 | 85.7 | 8627 | 539 | 5737 | 62.1 | 356 | 4734 | 81.1 | 293 |
| 2001 | 1621387 | 74.7 | 86.9 | 8809 | 550 | 5915 | 63.1 | 369 | 5012 | 83.4 | 312 |
| 2002 | 1627172 | 74.8 | 87.8 | 9001 | 562 | 6059 | 63.6 | 379 | 5176 | 85.2 | 324 |
| 2003 | 1630949 | 74.8 | 88.3 | 9190 | 574 | 6121 | 63.4 | 384 | 5249 | 85.2 | 329 |
| 2004 | 1632694 | 75.1 | 88.5 | 9364 | 586 | 6277 | 64.1 | 395 | 5451 | 86.4 | 342 |
| 2005 | 1635787 | 75.4 | 88.9 | 9551 | 599 | 6447 | 65.0 | 407 | 5564 | 85.8 | 351 |
| 2006 | 1637878 | 75.9 | 89.8 | 9740 | 611 | 6516 | 64.9 | 412 | 5685 | 86.6 | 360 |
| 2007 | 1642337 | 76.7 | 91.2 | 9913 | 622 | 6671 | 65.6 | 423 | 5955 | 88.6 | 378 |
| Men |  |  |  |  |  |  |  |  |  |  |  |
| 1992 | 1607532 | 77.5 | 81.3 | 10460 | 631 | 7773 | 67.2 | 459 | 6498 | 85.2 | 383 |
| 1993 | 1613078 | 76.3 | 75.8 | 10624 | 640 | 7727 | 65.7 | 456 | 5924 | 78.0 | 348 |
| 1994 | 1618450 | 75.2 | 75.2 | 10821 | 652 | 7683 | 63.8 | 453 | 5526 | 73.0 | 324 |
| 1995 | 1622976 | 75.1 | 77.0 | 11037 | 666 | 7735 | 63.1 | 457 | 5819 | 75.7 | 342 |
| 1996 | 1627020 | 75.2 | 77.7 | 11242 | 679 | 7852 | 63.3 | 466 | 5992 | 76.7 | 353 |
| 1997 | 1631477 | 75.4 | 80.0 | 11454 | 692 | 7960 | 63.2 | 474 | 6122 | 76.6 | 362 |
| 1998 | 1637109 | 76.3 | 82.9 | 11683 | 706 | 8182 | 64.2 | 489 | 6614 | 79.6 | 394 |
| 1999 | 1642867 | 77.2 | 84.6 | 11909 | 721 | 8497 | 66.1 | 511 | 7053 | 82.3 | 423 |
| 2000 | 1648016 | 77.6 | 86.1 | 12133 | 735 | 8708 | 66.9 | 536 | 7274 | 83.5 | 438 |
| 2001 | 1654268 | 77.9 | 86.8 | 12382 | 750 | 8941 | 67.9 | 542 | 7628 | 85.1 | 462 |
| 2002 | 1660538 | 77.7 | 86.8 | 12616 | 764 | 9012 | 67.5 | 548 | 7674 | 84.8 | 466 |
| 2003 | 1664299 | 77.5 | 86.9 | 12837 | 778 | 9114 | 67.5 | 555 | 7784 | 85.5 | 474 |
| 2004 | 1666171 | 77.6 | 87.2 | 13077 | 795 | 9219 | 67.4 | 564 | 7890 | 85.4 | 482 |
| 2005 | 1669523 | 77.9 | 87.9 | 13311 | 810 | 9398 | 68.0 | 577 | 8122 | 86.3 | 499 |
| 2006 | 1671743 | 78.2 | 89.2 | 13512 | 824 | 9603 | 68.8 | 591 | 8338 | 86.8 | 514 |
| 2007 | 1676540 | 78.7 | 90.7 | 13758 | 839 | 9786 | 69.0 | 604 | 8715 | 88.9 | 538 |
