# Supplementary material for: Working people with type 1 diabetes in the Finnish population
Source: BMC Public Health. 2017 Oct 12;17:805. doi: 10.1186/s12889-017-4723-8 (PMC5639775; doi:10.1186/s12889-017-4723-8)
Supplement: Supplementary file 3 — Characteristics of the participants in the “People with Type 1 Diabetes in Worklife” survey. (DOCX 14 kb) [file 12889_2017_4723_MOESM3_ESM.docx]

Additional file 3: Table S3 Characteristics of the participants in the “People with Type 1 Diabetes in Worklife” survey

|  | Employees  (n=767) |
| --- | --- |
| Gender (n=763) |  |
| Women | 337 (44.2) |
| Men | 426 (55.8) |
| Age, years (n=760) | 36.2 ± 12,4 |
| Marital status (n=766) |  |
| Married or cohabiting | 477 (62.3) |
| Unmarried, divorced, widowed | 289 (37.7) |
| Educational level (n=767) |  |
| Basic education (no education, only high school or vocational course) | 182 (23) |
| Vocational school | 264 (34.4) |
| Technical or vocational college | 217 (28.3) |
| University or university of applied sciences | 104 (13.6) |
| Length of employment, years (n=727) | 9.1±9.9 |
| Type of work (n=754) |  |
| Mental work | 311 (41.2) |
| Physical work | 190 (25.2) |
| Mental and physical work (equally) | 253 (33.6) |
| Duration of diabetes, years* (n=764) |  |
| 0–5 | 186 (24.3) |
| 6–10 | 249 (32.6) |
| 11–15 | 291(38.1) |
| > 16 | 38 (5.0) |
| HbA1c level (%)*(n=757) |  |
| ≤60 mmol/mol (≤7.5%) | 255 (33.7) |
| 61–70 mmol/mol (7.6–8.5%) | 261 (34.5) |
| 71–80 mmol/mol (8.6–9.5%) | 176 (23.2) |
| ≥81 mmol/mol (≥9.6%) | 65 (8.6) |
| Severe hypoglycemia*(n=761) |  |
| None | 587 (77.1) |
| Once | 89 (11.7) |
| Two or three times | 53 (7.0) |
| More often | 32 (4.2) |

Data are n (%) and mean ± SD.

* Self-reported
